# Infant-carrying mechanisms in a natural environment: the case of Qashqai nomad

**DOI:** 10.1017/ehs.2024.25

**Published:** 2024-10-30

**Authors:** Zohreh Anvari, Gilles Berillon, Kristiaan D'Août, Dominique Grimaud-Hervé, Mahtab Rezaei

**Affiliations:** 1 Social Science Faculty, Tehran University, Tehran, Iran; 2 UPR2147, CNRS, Paris, France; 3 Department of Musculoskeletal Biology, Institute of Ageing and Chronic Disease, University of Liverpool, UK; 4 UMR 7194, Muséum National d'Histoire Naturelle, Paris, France; 5 Biology Faculty, Tehran University, Tehran, Iran

**Keywords:** Infant-carrying, nomads, women, kinematics, kinetics

## Abstract

Infant carrying and more generally load carrying may impact bipedal locomotion and thus the energy cost of the daily activities, in living people but also in our ancestors. In order to improve our knowledge of infant carrying strategies we investigate the biomechanics of infant carrying in a non-mechanised group. The Qashqai are nomadic people who still carry loads and infants habitually without any daily assistance in varied natural environments. Our analysis focuses on the sagittal kinematics using a high-speed camera (joint angles, speed, position of the centre of mass) and kinetics (ground reaction forces and displacement of the centre of mass) using a six-degree of freedom force plate. We assessed the unloaded and loaded (infant) walking of 26 Qashqai women, living in the Fars province (Iran). The results demonstrate that different mechanisms of walking exist that are related to the mode of carrying and the weight of the infant, by which step length, walking speed and the lower limb angles are not affected. The displacement of the total centre of mass remains unchanged. This supports the hypothesis that the Qashqai have developed mechanisms of load carrying that limit the increase in energy consumption. This could be related to the usual high level of daily activity.

## Graphical abstract

**Figure:**
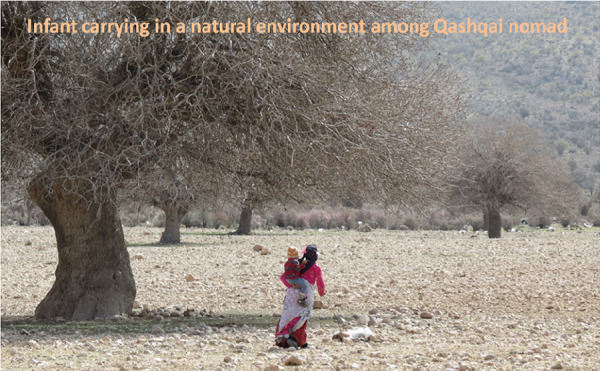

**Social media summary:** Infant carrying mechanics in Qashqai women reveal how load impacts energy efficiency and walking in natural contexts.

## Introduction

Load carrying in humans has been studied from varied perspectives, non-mechanised (they usually do not use or involve machines or automatic devices in transport and daily life) or mechanised peoples living in varied environments. Studies on Nepalese porters (Bastien et al., 2005), African pygmies (Jones et al., 1994) and women of the Kikuyu tribe (Maloiy et al., 1986) have investigated the capacity of living humans to carry heavy loads in their daily activities. For instance, African Pygmies, in comparison with European city dwellers, can carry relatively heavier loads without expending further metabolic energy (Jones et al., 1994). The oxygen consumption rate in Nepalese porters and Kikuyu women does not increase during carrying heavy loads in comparison with Europeans (Bastien et al., 2005; Maloiy et al., 1986). Kikuyu women can carry loads up to 20% of their body weights without an increase in their energy consumption (Maloiy et al., 1986). These studies have suggested that the level of experience as well as some training and/or anatomical adaptation since childhood, as well as unchanged stride frequency, could contribute to preventing an increase in energetic cost while carrying a heavy load. However, the precise mechanisms involved remain unknown. Indeed, testing these hypotheses requires biomechanical experiments in the field which have not been conducted so far. Load carrying has also been studied from an evolutionary perspective, in order to address the selective pressures that led to the adoption of permanent bipedalism in early hominins (e.g. Washburn, 1967; Fleagle, 1999). The reduction in body hair (Ruxton & Wilkinson, 2011) and the decrease in foot gripping ability (e.g. McHenry & Jones, 2006) as well as the increase in infant weight relative to that of the mother (Alemseged et al., 2006; Desilva, 2011) may have required new strategies, including the use of hands. These evolutionary traits may have made infant carrying more energetically costly in bipedal posture even though early hominins travelled shorter distances compared with later hominids (Kramer, 1998). The use of apparatus for infants carrying, which is believed to have begun 15,000 years ago, has also been addressed based on laboratory experiments with urban people (Wall-Scheffler et al., 2007).

More generally, the biomechanical impact of load carrying has been studied thanks to laboratory experiments on urban people (Tilbury-Davis & Hooper, 1999; Harman et al., 2000; Holt et al., 2003, Watson & Payne, 2008; Martin & Nelson, 1986; Obusek et al., 1995; LaFiandra et al., 2003; Hudson et al., 2021; Wang et al., 2021). These studies show that different strategies are developed. For instance, step length (the distance covered by an individual from the heel strike of one foot to the subsequent heel strike of the same foot in one step) is reduced when carrying loads heavier than 50% of body weight at high speeds (Martin & Nelson, 1986). However, at low speeds, load carrying does not affect step length (Obusek et al., 1995; Harman et al. 2000). Others have shown that load carrying at high speed reduces pelvic and thoracic rotation, which is responsible for controlling step length; the reduction in pelvic rotation would allow a decrease in the torque generated by the pelvis (LaFiandra et al., 2003). Holt et al.'s (2003) study on backpack carrying concluded that the load affects step length and step frequency. Step frequency, also known as step rate or cadence, refers to the number of steps taken per unit of time during walking or running. It is typically measured in steps per minute (spm). Step frequency is an important spatiotemporal parameter that provides information about the speed and efficiency of gait.

Load carrying may also impact the position and the trajectory of the centre of gravity (CoM) of the body during walking (e.g. Tilbury-Davis & Hooper, 1999). Even small changes in contact pattern or oscillation of the CoM can have potentially profound effects on energy consumption (Schmitt, 2011). However, various strategies of load carrying have been developed and seemingly reduce the oscillations of the CoM (see Table 1 for a synthesis). For instance, Wu and MacLeod (2001) show that, while carrying a load, the CoM shifts toward the load. However, this shift in CoM is smaller than the theoretical values because of a spatial reorientation of the pelvis and lower extremities, while maintaining the upper body orientation relatively unchanged. This strategy may allow increased stability of the upright posture with a minimal amount of muscle activation (Wu & MacLoad, 2001). In addition, Holt et al. (2003) have shown that an increase in musculo-skeletal stiffness allows maintaining a constant vertical excursion of the CoM across speed while carrying a load. Similarly, despite a decrease in joint flexibility, joint angles increase in the sagittal plane around the hip, knee and ankle, as well as the rotation of the CoM during load carrying (Harman et al.; 2000).

**Table 1.** Various load carrying positions and their impact on kinematic, kinetic and energetic aspects in urban and non-urban populations

	Heavy load on the back		Heavy load on the one side	
Load position		Non-urban	Urban	Non-urban
Spatiotemporal parameters	Affected	?	Affected	?
Angles	Increase in joint angles in the sagittal plane around the hip, knee and ankle	?	Increase in joint angles in the sagittal plane around the hip, knee and ankle	?
Oscillation of the CoM	Reduced vertical and lateral oscillations of the CoM	?	Reduced vertical and lateral oscillations of the CoM	?
Normalised GRF	Increased	?	Increased	?
Energy consumption	With expending further metabolic energy	Without expending further metabolic energy	?	?

?, Addressing the research question.

CoM, Centre of mass.

Finally, based on ground reaction forces (GRF) measurements, Tilbury-Davis and Hooper (1999) show that load carriage up to as much as 64% of the subject's body weight has little effect. For example, in trained subjects, increasing load carriage in backpacks raises ground reaction forces in proportion to the total mass (body mass plus mass of the carried load). The forces necessary for balance increase significantly when any load is carried; yet higher loads do not cause further significant increases in impact forces. Some researchers have pointed to an increase in GRF and impulse during loaded walking (Kinoshita, 1985; Harman et al., 2000; Birrell et al., 2007). Hsiang and Chang (2002) also reported an impact on the general bimodal pattern of GRF of load carrying: ventral and dorsal load carrying are associated with an increase in the first force peak and a decrease in the second peak.

In summary, this brief overview highlights that load carrying, and potentially infant carrying, may impact different aspects of posture and locomotion in humans. This impact may depend on the carrying modalities and the shape and the weight of the load itself, but also on the human population used for experiment, their origin and their habits of carrying loads. Despite this large framework, this field lacks referential biomechanical data for people living in varied natural environments and used to carrying loads without technical assistance. In the current study, we explore the impact of infant carrying on walking in a group of tribal women used to living in non-artificial environments, the Qashqais. By comparing the biomechanics of loaded and unloaded walking in a non-mechanised group, we aim at improving our knowledge of the fundamental mechanisms of load-carrying.

## Materials and methods

### Subjects

The Qashqai tribe represents one of several nomad tribes in Iran among other ethnicities (the Lores, Arabs, Torkamans, Kurds and Baluches; see Asgari Khaneghah & Kamali, 1995). The nomadic lifestyle extends to about 1.65% of the current Iranian population (Iranian Center for Statistics, https://old.sci.org.ir/english). The Qashqais migrate twice a year in the province of Fars looking for pasture for their sheep. Their travel distance is between approximately 30 and 730 km and can last for 40 days. The Qachqai's wintering area extends along the Persian Gulf to the south of the province at an average altitude of 450 m asl. Their summer district is the northern part of the province in the Zagros mountains; the altitude ranges from 2200 to 3943 m asl. In most cases, Qashqais do not use motorised vehicles to travel and their equipment and facilities are non-mechanised. The women are very active and have important roles, collecting and carrying wood, fuel, water and mountain plants and also taking care of their infants during migration and settlement. Families usually live separately and are spread over a vast area. We collected our data in their summer neighbourhood during a half-day approximately by family. The total time of observation lasted 6 months. Prior to the experiments, individual data were collected using a questionnaire including their age, the number and age of each of their children and the names of their summer and winter neighbourhoods in order to calculate the distance traveled during migration and the methods of travel (on foot, on the back of animals, by car, etc.), the type, duration and frequency of daily work, information on physical conditions, domestic activities and physical activity (searching for wood, water, etc.), nutrition, namely the components of theit diet (vitamins, minerals, proteins, etc.), and their potential musculoskeletal disorders and use of medications.

In total, we studied 26 women of this group and their infants; infants were between 50 days and 3 years old at the time of the experiment (Supplementary Information, SI, 1). For ethical reasons, numbers have been used instead of the names of the samples. All 26 women were used to travelling on foot, healthy and had no skeletal disease.

### Experimental setup

The principles of analysis and the experimental protocol were designed for the field conditions. The tools used had to be mobile and usable in non-artificial environments: a portable wooden walkway (4 m in length by 0.6 m in width when assembled) and a spatial calibration frame, a mobile force plate powered by solar panels and a rechargeable battery, a laptop and an autonomous video camera. After a short training period, we asked women to walk barefoot at constant speed on the horizontal walkway. (They are not habitual barefoot walkers but we asked them to carry their children without shoes and using cloth in order to explain the situation of early humans who lacked any means of transportation such as cloth to tie the child to themselves and shoes to walk.) In order to measure the dimensions of the space and to prevent parallax issues, the whole space was calibrated using 3D calibration.

The sagittal kinematics were recorded using a field motion analysis system made from a high-speed (120 images/s) and high-resolution video camera with a manually adjustable zoom (Canon PowerShot SX40 HS W4). The camera was positioned perpendicularly to the long axis of the walkway at a distance of about 3.5 m and 80–100 cm above the ground. Attempts were recorded on the camera's memory card in MVI format and exported onto an external disk in AVI format for further analysis.

### Load type and style

The Qashqai women carry various types of loads, including infants, water and wood for fuel. In this article, we focus on infant carrying. They carry their infants during migration and settlement periods from birth until infants are able to walk independently. On average, the infants are carried during migration and settlement periods without mechanical or technical assistance until they are around 5 years. In the settlement period, young infants usually stay in a tent with their grandmother or aunts and are occasionally carried by their mother.

**Figure 1.**
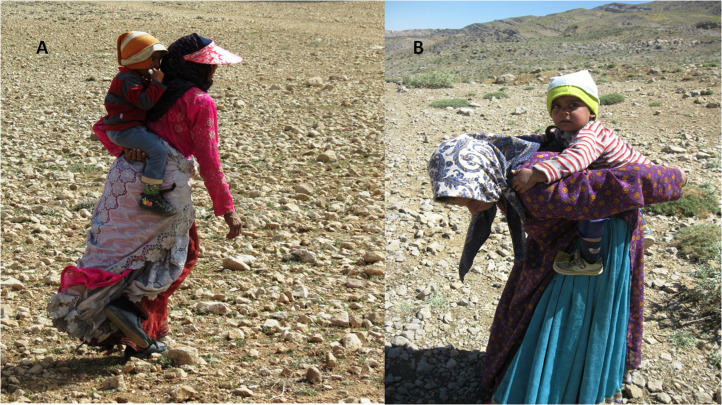
Qashqai women carrying baby in different types. (a) Symmetrically loaded; (b) asymmetrically loaded (photography by Zohreh Anvari).

Infant carrying by Qashqai women involved different techniques (Figure 1). Our observations during the migration and settlement periods and the results of interviews show that the Qashqai women use a symmetrically load (SL) type of carrying on the back more frequently than asymmetrically loading (AL) on the side. The SL allows some daily tasks to be performed for which both hands are required. The mother usually carries her baby on her back using a scarf from birth to one year old. Otherwise, she puts her first hand under the pelvis of the baby and with the second hand, she takes the baby's hand. When the infant gets older, the mother puts one or two hands under the infant's pelvis while the infant grips the mother's shoulders. Women use this mode of infant carrying during both migration and settlement periods. When the infant is rather heavy and older, he/she grips his/her mother's arms from behind. In AL, the mother carries the infant on one side of her body on the arm or on her hip. Qashqai women tend to carry their very small infants using this mode. Generally, the AL in Qashqai populations is seen during the settlement periods rather than during the migration periods. Mothers use this mode provisionally when there is no need to use both hands to conduct a task. This mode is dominantly used when the infant is rather lightweight (~9 kg) and is not yet old enough to hold on to his/her mother from behind while being carried.

According to our methodology, we asked the women to carry the infant on the walkway as they were used to doing. The women thus selected the type of infant-carrying. Women mainly carried their own children except for four women who carried the children of others. In total, the experiments allowed 343 sequences to be recorded from 26 women who walked on the walkway with and without infants (see SI 1 for the whole inventory). For the quantitative kinematic analysis and comparisons, only gait cycles performed strictly at constant speed and parallel to the long axis of the walkway were selected. They represent 182 of the 343 sequences and were made by 18 women: 73 unloaded sequences (UL), 40 AL sequences (only the right-side sequencesare visible: 18 at the arm and 22 on the hip) and 59 SL sequences (SI 2).

### Spatiotemporal parameters

Each walking cycle starts with the initial ground contact of a foot and ends with the next ground contact of the same foot. The cycle is divided into several successive phases defined by these events: initial foot contact, opposite foot take-off, opposite foot contact, take-off of the foot and final foot contact. The following spatiotemporal parameters were calculated: step length (the distance between the feet while both are in contact with the ground), stride length (m), stride duration (s), stride frequency (Hz), absolute speed (m/s, equal to the ratio between the stride length and the stride duration) and the duty factor (the amount of time that each extremity is in contact with the ground as a percentage of the total cycle duration, see Alexander, 1992). To reduce the effect of individual size, the absolute speed, stride length and stride frequency were made dimensionless. Dimensionless variables are a way to normalise or scale raw data in order to remove the units and make the data comparable across different scales. The relationship between dimensionless variables and raw data is that the dimensionless variables are derived from the raw data through a process of normalisation or scaling. This allows for easier comparison and analysis of the data, particularly when dealing with data that have different units or scales. Here the spatiotemporal data were recalculated to dimensionless quantities according to the principle of dynamic similarity. Lower leg length was used as the reference length (Aerts et al., 2000).

### Anatomical model, joint and limb angles

Each individual is represented by a multi-segment model made up of 23 anatomical points on the mother's body and 13 anatomical point on the infant's body. For the mother, points are the following: six points on visible posterior limb comprising the end of the foot (third ray), the metatarsophalangeal joint (fifth ray), the heel (calcaneus), the ankle (lateral malleolus), the knee (patella) and the hip (trochanter major); four points on the opposite posterior limb including the ankle (malleolus medialis), the heel (calcaneus), the knee (patella); 14 points on the visible anterior limb including the end of the hand (third ray), the wrist (middle), the elbow (lateral epicondyle) and the shoulder (acromion); three points on the non-visible opposite anterior limb comprising the end of the hand (third ray), the wrist (middle), the elbow (olecranon); two points on the trunk comprising the lumbosacral joint, base of the head (occiput); two points on the head comprising the vertex and chin. Women's traditional clothing can hinder the acquisition of anatomical points. In order to respect this wearing of the traditional clothing and ensure the accuracy of the measurements, we placed elastic bands with markers over the clothing at the location of the joints. We also put the markers on the elastic band to highlight the knee, elbow and trochanter points. Finally, despite the use of elastic bands, the clothes sometimes obstructed the observation of the points, which led us to exclude certain sequences from the analysis.

For the infant, the points are the following: three points on the visible anterior limb comprising the wrist (middle), the elbow (lateral epicondyle) and the shoulder (acromion); three points on the visible posterior limb comprising the ankle (malleolus laterale), the knee (patella) and the hip (trochanter major); two points on the opposite anterior limb comprising the wrist (middle) and the elbow (lateral epicondyle); two points on the opposite posterior limb comprising the ankle (malleolus laterale) and the knee (patella); and three points on the head comprising the vertex, the base of the neck and the chin.

Each anatomical point was digitised manually, frame by frame, using Kwon3D software (Visol). The digitised points and their coordinates were calibrated and converted to Cartesian coordinates through a calibration procedure. This procedure involved the calibration frame (1.7 m long and 1 m in height and width) and was performed with the KwonCC calibration module. The average reconstruction error for the calibration files was 2.17 cm. From these coordinates, we were able to calculate the angles between segments at the ankle, knee, hip and metatarsophalangeal joint and the angle of inclination of the trunk. The mean values and standard deviation at each event and the graphic representations of their pattern during a gait cycle were used to compare loaded and unloaded gait parameters.

### Kinetic analysis

Ground reaction forces (*Fx*, *Fy*, *Fz*) were recorded using a force plate (Kistler 9260AA3) at 200 Hz, placed in middle of the walkway and based on a stabilised horizontal zone. The signals were transferred to the computer via a National Instuments USB DAQ board (type USB-6008) and read using LabView software developed by one of us (KD). This application allows the acquisition of the raw data (V), their conversion into newtons, their visualisation and then their export in text format.

We calculated the vertical force variables according to Hsiang and Chang (2002): the first peak (P1); corresponding to the weight acceptance; and the second peak (P2) corresponding to the propulsion phase and the minimum point between the two maxima (M) corresponding to the middle of the stance phase (Allard & Blanchi et al., 2000; Viel, 2000). We calculated the Impulse (N.s) using the following formula (Kimura, 1992):
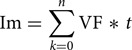
where Im is the vertical impulse (N.s), VF is the vertical force (N) and *t* is the duration of the interval between each force value (s).

There were 267 force sequences that were selected for 26 individuals: 128 unloaded, 95 loaded on the back and 44 lateral load sequences.

### Centre of mass

Based on Newton's second law, we calculated the displacement of the centre of mass (CoM) during the single support phase using the ground reaction forces recordings according to the following formula:
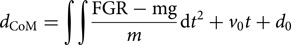
where *d*_CoM_ is the displacement of the centre of mass; FGR is the ground reaction force, *mg* is the weight, *m* is the mass, *t* is the time, *v*_0_ is the displacement speed of the centre of mass and *d*_0_ indicates the initial location (Bonnefoy-Mazure & Armand, 2015).

The GRF between the first peak and the second peak was used to calculate the *d*_CoM_ during the single support phase; the opposite leg does not contact the ground and the corresponding GRFs are thus nil.

The total CoM excursion could be estimated thanks to the kinematic model. The CoM is defined as the centroid of *n* segments on the *x*-axis according to the following formula:
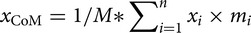
where *x*_CoM_ is the position of the total centre of mass, *M* is the body mass, *m_i_* is the segmental mass and *x_i_* is the position of the segmental centre of mass (Gutierrez-Farewik et al., 2006).

We know the body mass of the mothers and those of the infants. Their segmental properties (CM% and mass) were estimated using the following methods and implemented by the Kwon software (BSP). According to Shan and Bohn (2003) we estimated the segmental mass from the total mass and size of body of each individual as follows:
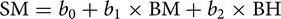
where SM is the segmental mass, BM is the body mass, BH is the body size and *b*_0_, *b*_1_ and *b*_2_ are the coefficients provided by Shan and Bohn (2003) for their German and Chinese women sample.

For infants, we used the formula of Van Dam et al. (2009):
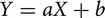
where *Y* is the segmental mass, *X* is the body mass and *a* and *b* are the coefficients calculated for each segment by Van Dam et al. (2009).

You can see the average segmental centre of mass as a percentage of total body mass (CM%) for each segment, for the mothers and the infants respectively in SI 3 and 4.

Finally, we calculated the position of the total centre of mass and its displacement for unloaded (mother only) and loaded (mother and infant) conditions and then estimated the vertical elevation of the CoM (Δ*h*) for each condition. In one stride, the position of the CoM is at its lowest at the time of contact and take off of the foot, and at its highest at mid-stance (Heglund et al., 1995; Schmitt, 2011). The distance between the lowest and the highest elevations of the CoM is represented by Δ*h*.

### Statistics

Differences were tested using covariance analysis (ANCOVA, Statistica, 6.0). For the angles, the angle is used as the dependent variable, the type of gait (loaded vs. unloaded) as the independent variable and the dimensionless speed as the covariate. For the force parameters, the force values (the maximum peaks and minima) are used as dependent variables, the type of gait (loaded vs. unloaded) as the independent variable and the dimensionless speed as a covariate. The loaded and unloaded spatiotemporal parameters were compared using a *t*-test. Force parameters and the standardised elevation of total centre of mass (Δ*h*) were compared using ANOVA (Statistica, 6.0) and an independent *t*-test (SPSS statistics, 22).

## Results

### Infant positioning

Table 2 summarises the number of different types of infant carrying sequences, the percentage of body weight (%BW) and the percentage of different support types (1S, 2S). Increasing the %BW does not affect the percentage of different support types significantly.

**Table 2.** The percentage of body weight (%BW), the percentage of different support types (1S, 2S) and the mean of spatiotemporal parameters ± standard deviation (SD) of a walking cycle of unloaded women (UL) and women carrying their infant with symmetrical load (SL) and asymmetrical load (AL) models

Individual	Type	N	%BW	2S	1S	L(m)	Ld	D(s)	Dd	Sa(m/s)	Sd	DF
Mean	UL	76	0	28.96 ± 4.19	71.03 ± 4.2	1.18 ± 0.13	2.71 ± 0.37	1.04 ± 0.09	0.50 ± 0.05	1.13 ± 0.14	0.54 ± 0.06	0.64 ± 0.07
Mean	AL	43	22.59	30.15 ± 3.55	69.8 ± 3.58	1.05 ± 0.13	2.53 ± 0.33	1.02 ± 0.11	0.50 ± 0.07	1.04 ± 0.19	0.51 ± 0.08	0.65 ± 0.02
*p*				**0.1**	**0.1**	**0**	**0**	**0.3**	**0.7**	**0.003**	**0.02**	**0.2**
Mean	SL	59	24.01	29.14 ± 4.36	70.82 ± 4.38	1.18 ± 0.16	2.72 ± 0.3	1.1 ± 0.15	0.53 ± 0.09	1.09 ± 0.22	0.52 ± 0.09	0.64 ± 0.03
*p*				**0.8**	**0.7**	**0.6**	**0.8**	**0.004**	**0.009**	**0.1**	**0.1**	**0.5**

*p-*Values < 0.05 from the *t*-test. Bold indicates a significant difference ≤0.05 with the *t*-test. Spatiotemporal parameters include the absolute (*L*, m) and dimensionless (*Ld*) stride length, the absolute (*D*, s) and dimensionless (*Dd*) stride duration, the absolute (*Sa*, m/s) and dimensionless (*Sd*) speed and duty factor (DF).

### Spatiotemporal parameters

The average values of the spatiotemporal parameters are given in Table 2. These parameters include the absolute (*L*, m) and dimensionless (*Ld*) stride length, the absolute (*D*, s) and dimensionless (*Dd*) stride duration, the absolute (*Sa*, m/s) and dimensionless (*Sd*) speed and duty factor (DF).

In asymmetrically loaded conditions, the stride length and speed decrease compared with the unloaded condition while the stride duration does not differ. In symmetrical carrying, stride duration increase compared to unloaded conditions, while the stride length and speed do not change (Table 2). With regards to the %BW, speed is not affected as a function of %BW whatever the loaded condition. Stride length and stride duration increase as a function of %BW in asymmetrically loaded conditions. In the symmetrically loaded condition, stride length increases and stride duration decreases.

Duty factors are not affected by infant carrying, whatever the loaded condition, compared with the unloaded condition (Table 2). Increasing the %BW does not affect the duty factor when loaded.

### Joint angle profiles

**Figure 2.**
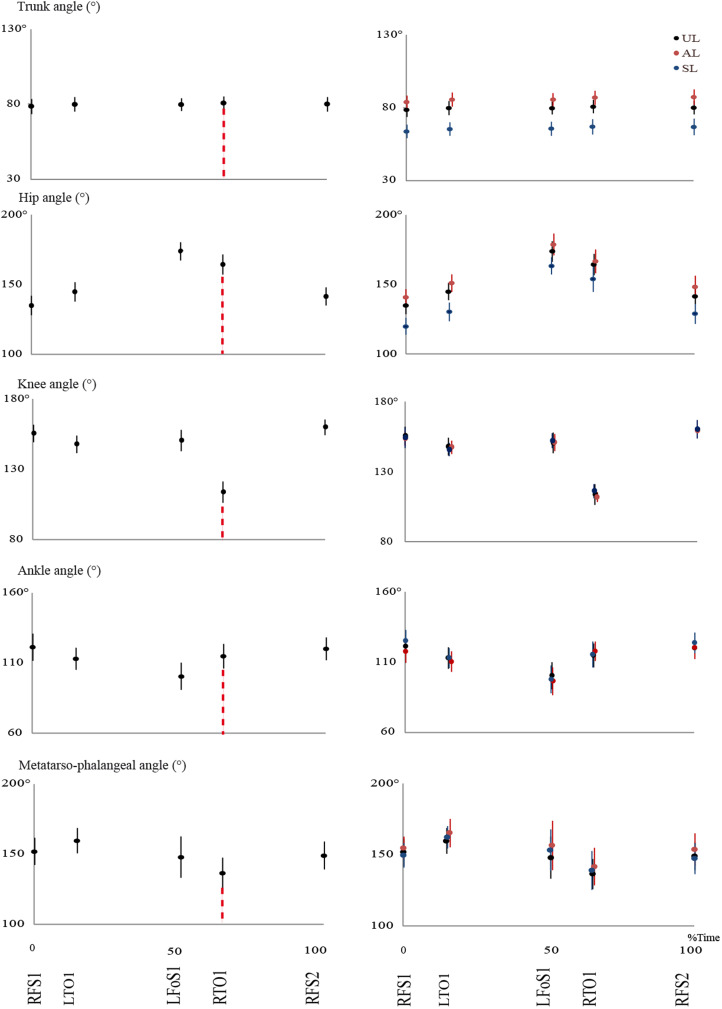
Comparison of mean changes (mean ± SD) of the trunk, hip, knee, ankle and metatarsophalangeal angles and events through the bipedal stride of unloaded (black symbols) and loaded (symmetrical, SL, blue symbols; and asymmetrical, AL, red symbols) Qashqai women. FSI, Initial foot contact; LTO1, opposite toe-off; LFoS1, opposite foot contact; RTO1, toe-off (arrow: TO); RFS2, final foot contact.

**Figure 3.**
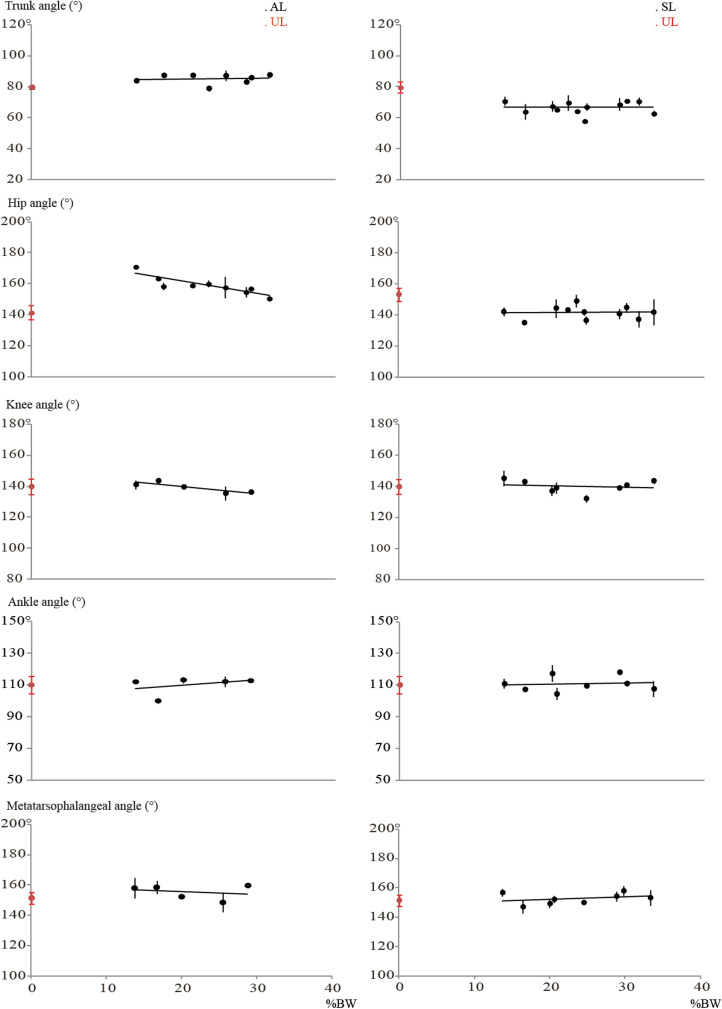
Average angle (degrees) as a function of increasing the infant mass (%BW) in unloaded (UL) and loaded (symmetric, SL; and asymmetric, AL) position.

Figure 2 summarises the changes in trunk, hip, knee, ankle and metatarsophalangeal angles for loaded and unloaded walking and their variation over time expressed as a fraction of the cycle (see SI 5 for more details). The average angles as a function of %BW are given in Figure 3. In AL, the trunk is more erect and the hip is more extended than in the UL condition. No differences are observed for the knee, the ankle and the metatarsophalangeal angles. As shown in Figure 3, in the AL condition, the hip angle decreases as a function of %BM, while the trunk, the knee, the ankle and the metatarsophalangeal angles do not change.

In the SL condition, the trunk is more inclined forward and the hip is more flexed compared with the UL condition. The knee, the ankle and the metatarsophalangeal angles are not affected by infant carrying. Increasing the %BW has no effect on any of the joint angles in SL position.

### The vertical force values

**Figure 4.**
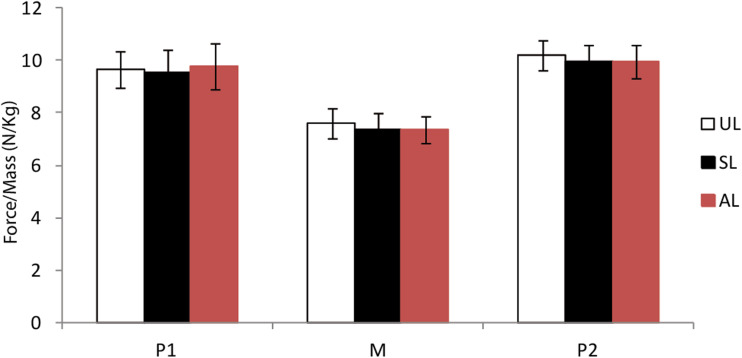
Comparison of the average (±SD) of the first peak (P1), the second peak (P2) and the middle of stance phase (M) of the force profile in unloaded (UL) and asymmetrically (AL) and symmetrically loaded (SL) conditions.

Figure 4 compares the averages and the statistical data of the vertical force variables comprising the first peak (P1), the second peak (P2), the average of the stance phase (M) and the standardised impulse (Im) by the total mass (mother and infant) for different %BW.

When carrying asymmetrically, P1 increases with 15%BW, M and P2 decrease with 20%BW and P1 and P2 decrease with 25%BW significantly. During symmetrical carrying, P1 increases with 15%BW, P2 decreases with 20%BW, M decreases with 25%BW and M and p2 decrease with 30%BW. The normalised average impulse decreases significantly in the loaded (AL, SL) positions in compare with the unloaded position (see SI 7 and 8 for more details).

### The centre of mass

In both loaded conditions, *d*_CoM_ does not change significantly compared with the UL condition (Table 3). In addition, the %BW (significance 0.488), the carrying position (significance 0.865) and both of them (significance 0.958) do not impact the *d*_CoM_.

**Table 3.** : Average values, Standard Deviation (M±SD) and values of t test (p values <0.05) of the displacement of the centre of mass (dCoM) according to different %BW in unloaded (%BW=0) and symmetrical loaded (SL); and Asymmetrical loaded (AL). N is the Number of sequences.

%BW	N SL		dCoM UN/SL	N AL	dCoM UN/AL
0	115	M±SD	-.003±.012	115	-.003±.012
15	9	M±SD	-.001±000	13	-.001±.000
		p	-712		.644
20	24	M±SD	-.001±.000	9	-.001±.000
		p	.564		.727
25	42	M±SD	-.003±.003	12	-.005±.004
		p	.761		.485
30	6	M±SD	-.003 ± 001	6	-.002 ± .001
		p	.976		.893

**Figure 5.**
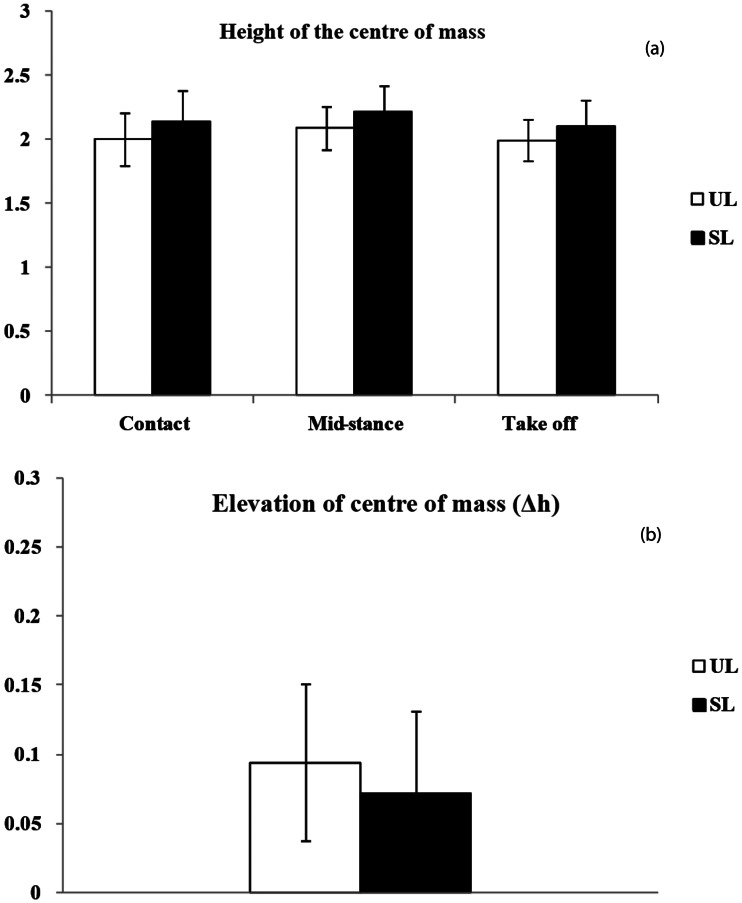
(a) Standardised height (±SD) of total centre of mass at the time of the contact, the middle of the stance phase and foot take-off. (b) Standardised elevation (Δ*h*) (±SD) of total centre of mass during the stance phase in unloaded (UL) and symmetrically loaded (SL) conditions.

Figure 5 presents a summary of the height (*Y*) and elevation (Δ*h*) of the total CoM for the unloaded and symmetrical loaded conditions. In the SL condition, the CoM is higher compared with the UL condition. This difference is significant in total and when the women carry an infant with 20%BW. Although the standardised height of CoM (*Y*) increases at 20%BW, the standardised vertical CoM elevation (Δ*h*) decreases (see SI 6 for more details).

## Discussion

This study is among the few biomechanical studies on load carrying in contemporary non-mechanised human populations living in natural environments and is the only one on infant carrying in a nomadic population. The study focuses on 26 women and their infants (or the infants of others) to compare the kinematics and kinetics of walking in women carrying infants of different ages and body mass with those of unloaded walking. The biomechanical data presented include the spatio-temporal parameters, joint angles, infant position changes, oscillations of the centre of gravity of the body, ground reaction force and displacement of centre of mass. Both the infant's weight and their position may influence each of these parameters.

Research conducted in the biomechanical engineering lab, utilising a treadmill, has revealed that the method of carrying infants in humans is influenced by various factors such as the infant's weight and age, the mother's daily activities and lifestyle, and the cultural practices of the population (Singh, 2009; Watson et al., 2011). Observations from non-mechanised populations in Africa, Mexico, India, Thailand, Nepal (Singh, 2009) and Iran (Asgari Nuri Nik kholgh, 1998) indicate that women typically secure their infants on their backs while walking freely. In the current study, Qashqai nomadic women preferred this mode of infant carrying during both migration and settlement periods and during daily tasks for which both hands are required. This is particularly true when the infant is relatively heavy and old enough to grip his/her mother's arms from behind.

### Symmetrical infant carrying

In symmetrical infant carrying on the back, the mother's back is more inclined forward and consequently the angle of her hip is more flexed. According to Goh et al. (1998), this simple mechanism enables the mother to maintain her balance during load carrying on her back. The trunk is tilted forward and the infant's extra mass is located close to the mother's CoM. In fact, the back being inclined forward prevents any change in the centre of gravity and consequently enables the mother to maintain her balance. In this mode of infant carrying, there seems to be no change in the angles of the lower limbs, and the infant's increasing weight has no effect on angles of the lower limb's joints. Our study also indicates that infant carrying on the lumbar portion of the back causes the total centre of gravity (mother and her infant) to rise. This rise is accompanied by a decrease in the vertical oscillations of the centre of gravity, which is probably related to the fact that the inclinations of the lower limbs’ joints remain unchanged during infant carrying compared with unloaded walking.

These changes are also followed by a decrease in the rate of impulse and the peaks of vertical GRF. Researchers report an increase in GRF during load carrying, particularly when the joints angles are more flexed (Birrell et al., 2007), and the infant's posture during walking lead to these changes in vertical oscillations of the centre of gravity. In this position, owing to an increase in the vertical rotation of the centre of gravity, the rate of metabolic energy consumption usually increases (Wall-Scheffler et al., 2007; Watson & Payne, 2008; Holt et al., 2003). However, among African women, carrying loads up to 20% of the body weight does not affect their metabolic energy consumption rate (Maloiy et al., 1986; Heglund et al., 1995). Our findings suggest that in symmetrical carrying on the back, the vertical oscillations of CoM and the normalised vertical force parameters decrease and *d*_CoM_ does not change, whereas there appears to be no change in the angles of lower limbs joints. Therefore, it can be assessed based on the previous studies that the lack of change in the angles of the lower limbs joints results in a decrease in the vertical oscillations of the centre of gravity and consequently the GRF.

The results of our study suggest that in infant carrying on the mother's back, walking speed and step length do not change significantly (see Table 2). In this position, the infant's increasing weight does not alter walking speed and duty factor, but the step length increases and the step duration decreases. LaFiandra et al. (2003) found that a high, thoracic location of the load (in their case, a backpack) leads to a decrease in pelvic rotation and step length. Conversely, a low, pelvic location of the load (in our case, an infant) might have a minimal impact on pelvic rotation and on step length. This enables the Qashqai women to maintain their walking speed and step length during infant carrying on the back.

### Asymmetrical infant carrying

Asymmetrical infant carrying is conducted in two ways. First, the mother carries her infant on one side of her body – on the hip. This mode is common among some populations including those in Sri Lanka (Bruser, 1981) and our cases in the current study (the Qashqai women). Second, the mother holds the infant in her arms. This mode is frequently seen in urban settings and is less common in non-urban and non-mechanised populations. Qashqai women tend to carry only their very small infants using this mode. Generally, asymmetrical infant carrying in Qashqai populations is usually seen in settlement periods rather than migration periods. Mothers use this mode provisionally when there is no need for both hands to perform a task. This mode is dominantly used when the infant is light and not yet capable to grip his/her mother from behind during carrying.

In asymmetrical infant carrying, the back is straighter and the hip is more extended, whereas there is no evidence of change in the angles of the lower limbs joints (knee, ankle and the metatarsophalangeal joint). The infant's increasing weight is not accompanied by any change in the angle of the mother's back during carrying, while the angle of the hip decreases and the angle fluctuation of the lower limbs joints is observable. These changes are associated with some changes in different phases of the vertical forces: there is no change when the leading foot contacts the ground, but immediately the midstance and lifting of the foot (the second pick) decrease. The position of the infant during asymmetrical carrying may affect these changes. The hip angle is wider when load carrying with respect to not load carrying. When the foot contacts the ground during infant carrying, the total force (the mother's body and her infant) is on the leading foot (the same foot for which GRF is calculated) and the infant on this food prevents the hip from flexing. Most probably, this causes no decrease in the vertical oscillations of the centre of gravity in the initial contact. In the middle and at the end of the phase, with no change in the lower limb joints the vertical oscillations of the centre of gravity decrease and lead to a decrease in the force rate over the dominant foot. The *d*_CoM_ does not change in asymmetrical infant carrying. The walking speed and step length decrease significantly. Hip rotation caused by infant carrying may lead to these changes.

### Symmetrical vs. asymmetrical infant carrying

In summary, by comparing the two mechanisms of infant carrying, the preference of Qashqai women towards symmetrical load carrying on the back, particularly in migration periods, could be better understood. Meanwhile, since load and infant carrying are considered as parts of the Qashqai women's daily lives, they have learned by experience and/or by cultural transmission that the most effective body parts for infant carrying are either the lower part of the back or the hip. Besides, owing to high levels of physical activity during load carrying, there is no change in their angles of lower limbs joints. As such, the vertical oscillations of centre of gravity decrease and the *d*_CoM_ does not change

Our findings support those of Watson and Payne (2008) and Wall-Scheffler et al. (2007) illustrating the preference for a symmetrical model of infant carrying. These scholars examined the energy consumption rate of urban women during infant carrying. They tried to answer the palaeoanthropological questions regarding long-distance infant carrying in early humans without the aid of tools (up to 15,000 years ago).

Therefore, our findings along with others on the biomechanics of walking among African women (Maloiy et al., 1986), Nepalese porters (Bastien et al., 2005) and members of populations who walk and run with bare feet (D'Aout et al., 2009; Hatala et al., 2013) highlight the need to investigate the non-mechanised populations in order to obtain a broader understanding of human variety. Populations living in natural settings are more appropriate for paleoanthropological studies than urban cases, because of their life conditions in terms of feeding habits (substantial amounts of dairy products), high levels of physical activity and minimum use of mechanised tools in their daily tasks. The Qashqai nomads are almost not impacted by artificial environments.

Our kinematic and kinetic research on loaded walking in non-mechanised people and those which are mainly conducted on urban cases in laboratories on treadmills (Kinoshita,^1985^; Harman et al., 2000; Hsiang and Chang, 2002; Watson et al., 2009; Wall-Scheffler, 2022) have yielded different results. Studies on urban populations concluded that there is: a decrease in walking speed during load carrying; inclination and consequently a decrease in the angle of the lower limbs joints which leads to an increase in the vertical oscillations of centre of gravity; and an increase in GRF rate over the dominant foot during load carrying. For example, Kinoshita (1985) argues that there is a decrease in ankle and knee angles of up to 40% of the body weight during load carrying. Watson et al. (2009) indicate that women carrying loads of up to 20% of their body weights on their hips show a significant ipsilateral flexion in their knees, but there is no evidence of any change in their hip angles. On the contrary, in Qashqai people, the results show: no significant change in the walking speed in SL; no change in the angle of the lower limbs joints and a decrease in the vertical oscillations of centre of gravity; no change in *d*_CoM_; and a decrease in GRF rate over the dominant foot during load (infant) carrying.

These original data suggest that usual physical activity in comparison with urban dwellers have enabled the Qashqai to prevent any change in the angle of the lower limbs joints. Similar findings by Tilbury-Davis and Hooper (1999) support this assumption. Tilbury-Davis and Hooper (1999) studied American soldiers who daily had to carry heavy swags on daily basis. The kinematic and kinetic data of this research, in comparison with other studies (Kinoshita, 1985; Harman et al., 2000; Birrell et al., 2007), yielded different findings. They also report no change in the angle of the lower limbs joints in trained soldiers with high levels of physical activity. Likewise, in their study, the GRF over the dominant foot was reported to be decreased during load carrying. Tilbury-Davis and Hooper (1991) assume that the difference between the results of their study compared with others may be due to the task experience and training of these soldiers in carrying heavy backpack.

## Conclusion

Infant carrying may impact different aspects of posture and locomotion in living humans. This impact may depend on the carrying modalities and the infant weight, but also on the human population used for the experiment, their origin and their habits of carrying loads. In the current study, we explore the impact of infant carrying on walking in a group of tribal women in a non-artificial field environment. As a result, Qashqai women appear to develop some suitable strategies, like selecting the best position of load/infant carrying on the body. The study supports the hypothesis that different mechanisms of walking exist that are related to the mode of carrying and the weight of the infant. When carrying an infant, step length and walking speed do not change, and the lower limb angles are not affected. The displacement of the total centre of mass remains unchanged which may help limit the energy cost of infant carrying. This could be related to a usual high level of daily activity.

Although such field experiments on non-mechanised people may be improved by future investigations, they prove the need for experimental data gathered in natural environments for our general knowledge of load-carrying strategies during bipedal walking.

## Supporting information

Anvari et al. supplementary material Anvari et al. supplementary material

## Data Availability

The datasets generated and analysed during the current study are available in the CNRS (France) and University of Tehran (Iran) repositories. Restrictions apply to the availability of these data, which were used under licence for the current study and so are not publicly available. Data are, however, available from the authors upon reasonable request and with permission from CNRS and University of Tehran.

## Supplementary material

The supplementary material for this article can be found at https://doi.org/10.1017/ehs.2024.25.

## Research transparency and reproducibility

These have been explained in the ‘Experimental setup’ section in the article.
