# Supplementary material for: Infant-carrying mechanisms in a natural environment: the case of Qashqai nomad
Source: Evol Hum Sci. 2024 Oct 30;6:e36. doi: 10.1017/ehs.2024.25 (PMC11588558; doi:10.1017/ehs.2024.25)
Supplement: Anvari et al. supplementary material [file S2513843X24000252sup001.docx]

**Supplementary Information (SI)**

| **Mother** | | | **Infant** | | | |  |
| --- | --- | --- | --- | --- | --- | --- | --- |
| **Individual** | **age (year)** | **Mass (kg)** | **Individual** | **Sex** | **age (year)** | **Mass (kg)** | **Number of sequences**  **UL/AL/SL** |
| 1 | 25 | 52 | Y | M | 1,8 | 15 | 5/0/3 |
| 2 | 26 | 52 | Y | M | 1,8 | 15 | 6/3/0 |
| 3 | 38 | 60 | Z | F | 2 | 11,5 | 5/0/3 |
| 4 | 28 | 51 | F | F | 2 | 12,5 | 4/0/3 |
| 5 | 30 | 52 | P | F | 0,58 | 7 | 7/3/2 |
| 6 | 28 | 48 | H | F | 3 | 15 | 5/2/3 |
| 7 | 31 | 71 | S | F | 0,91 | 15 | 8/0/7 |
| 8 | 28 | 51 | A | M | 0,75 | 8,5 | 7/4/0 |
| 9 | 30 | 65 | A | M | 0,75 | 8,5 | 7/2/2 |
| 10 | 35 | 51 | A | M | 0,75 | 8,5 | 11/0/7 |
| 11 | 26 | 49 | A | M | 0,75 | 8,5 | 5/7/0 |
| 12 | 28 | 52 | M | M | 1 | 11 | 5/7/6 |
| 13 | 40 | 43 | Sa | F | 1 | 10 | 5/8/13 |
| 14 | 40 | 52 | Aa | M | 1,5 | 11,5 | 6/0/6 |
| 15 | 30 | 52 | Al | M | 2,5 | 11 | 8/0/5 |
| 16 | 30 | 51 | Ho | M | 0,58 | 7 | 3/4/4 |
| 17 | 21 | 46 | Ay | F | 0,15 | 4,5 | 15/0/5 |
| 18 | 30 | 79 | Mo | F | 1,5 | 10 | 4/0/7 |
| 19 | 24 | 40 | Se | F | 1,4 | 8 | 6/4/4 |
| 20 | 40 | 60 | Fa | M | 0,75 | 7 | 4/2/11 |
| 21 | 30 | 51 | Mi | F | 1,5 | 10,5 | 4/1/6 |
| 22 | 32 | 51 | Ma | F | 3 | 17 | 5/0/8 |
| 23 | 39 | 63,5 | D | F | 2,5 | 14 | 11/0/6 |
| 24 | 35 | 73 | R | M | 2 | 12 | 5/0/8 |
| 25 | 19 | 58 | Ar | M | 1,5 | 11 | 7/5/0 |
| 26 | 29 | 49,5 | Ay | F | 2,5 | 12 | 9/0/8 |
| 26 Women | 30/46 | 54/7 | 22 Infant |  | 1.4 | 10.82 | 167/52/127 |

SI 1: Characteristics of Qashqai women and their infants. UL: unloaded sequences; SL: symmetrical loaded sequences (infant was carried on the back); AL: Asymmetric loaded sequences (the infant was carried on hip and at the arm, on the left or the right side).

| Individual | UL | SL | AL: Arm/Hip |
| --- | --- | --- | --- |
| 2 | 6 | 3 | 0 |
| 8 | 1 | 4 | 0 |
| 1 | 3 | 3 | 0 |
| 6 | 5 | 2 | 2/0 |
| 4 | 3 | 0 | 3/0 |
| 13 | 4 | 7 | 0/11 |
| 11 | 4 | 0 | 7/0 |
| 14 | 4 | 5 | 0 |
| 12 | 5 | 0 | 0/7 |
| 9 | 2 | 0 | 2/0 |
| 15 | 8 | 5 | 0 |
| 16 | 3 | 4 | 0/4 |
| 18 | 4 | 7 | 0 |
| 21 | 4 | 5 | 0 |
| 24 | 4 | 7 | 0 |
| 26 | 5 | 8 | 0 |
| 22 | 3 | 5 | 0 |
| 19 | 5 | 4 | 4/0 |
| Total | 73 | 69 | 18/22 |

SI 2: The selected video sequences by individual when unloaded (UL) and loaded (Asymmetrically, AL, and Symmetrically, SL).

| Segments | Proximal-Distal | CM% |
| --- | --- | --- |
| Head | VERT-BN | 41 |
| Trunk | BN-L | 47,08 |
| Forearm | E-W | 42,7 |
| Arm | SH-E | 45,1 |
| Hand | W-EXH | 37,6 |
| Thigh | GT-K | 32,55 |
| Leg | K-A | 45,8 |
| Foot | H-EXF | 47,05 |

SI 3: Position and the average segmental center of mass as a percentage of total body mass (CM%) for each segment of Women. VERT = Vertex, BN = Base of neck, L = Lumbosacral, E = Elbow, W = Wrist, SH = Shoulder, EXH = End of hand, GT = Grand Trochanter, K= Knee, A= Ankle, H= Heel, EXF = End of Foot (Shan et Bohn, 2003; modified)

| Segments | Point proximal - Point distal | CM% |
| --- | --- | --- |
| Head | Vertex-Cervical | 45,72 |
| Trunk | Cervical-Sacro coxal | 55,06 |
| Forearm | Shoulder - Elbow | 51,48 |
| Arm | Elbow - Wrist | 54,31 |
| Thigh | Hip - Knee | 54,8 |
| Leg | Knee - Ankle | 54,87 |

SI 4: Position and the average segmental center of mass as a percentage of total body mass (CM%) for each segment of infants (Van Dam et al., 2009 modified).

| **Angle** | **Type** | **N** |  | **IFC** | **OTO** | **OFC** | **TO** | **FFC** | **Range** |
| --- | --- | --- | --- | --- | --- | --- | --- | --- | --- |
| Trunk | UL | 76 | M | 78,73±4,93 | 79,95±4,79 | 79,85±4,35 | 80,83±4,55 | 80,2±4,71 | 5,42±2,67 |
|  | AL | 43 | M | 84,11±4,53 | 85,77±4,68 | 85,84±4,51 | 87,22±4,75 | 87,51±5,28 | 5,91±3,46 |
|  |  |  | P | ***0*** | ***0*** | ***0*** | ***0*** | ***0*** |  |
|  | SL | 59 | M | 63,86±4,57 | 65,51±4,65 | 65,86±4,76 | 67,18±5,19 | 66,97±5,67 | 5,38±3,4 |
|  |  |  | **P** | ***0*** | ***0*** | ***0*** | ***0*** | ***0*** |  |
| Hip | UL | 76 | M | 135.14±6.92 | 145.05±7.08 | 174.15±6.57 | 164.61±7.27 | 141.68±6.49 | 41.93 ±7.91 |
|  | AL | 43 | M | 141.01±5.75 | 151.28±6.39 | 178.97±7.85 | 166.95±8.32 | 148.53±8.13 | 40.25 ±6.76 |
|  |  |  | **P** | ***0*** | ***0*** | ***0*** | ***0,02*** | ***0*** |  |
|  | SL | 59 | M | 120.01±5.95 | 130.64±6.64 | 163.55±6.3 | 154.2±9.01 | 129.26±7,19 | 45.89±7.14 |
|  |  |  | **P** | ***0*** | ***0*** | ***0*** | ***0*** | ***0*** |  |
| Knee | UL | 76 | M | 155,8±6,43 | 148,15±6,21 | 150,75±7,45 | 113.92±7,42 | 160,3±5,48 | 61,95±7,57 |
|  | AL | 43 | M | 154,02±5,27 | 147,79±4,74 | 151,16±5,99 | 112,05±3,38 | 159,57±4,63 | 61,89±6,29 |
|  |  |  | **P** | ***0,1*** | ***0,3*** | ***0,79*** | ***0,59*** | ***0,73*** |  |
|  | SL | 59 | M | 154,58±7,33 | 145,94±4,76 | 152,3±5,31 | 116,5±5,09 | 160,53±6,72 | 59,12±5,88 |
|  |  |  | **P** | ***0,2*** | ***0,2*** | ***0,3*** | ***0,11*** | ***0,6*** |  |
| Ankle | UL | 76 | M | 121,62±9,81 | 113,3±7,69 | 100,69±9,63 | 115,14±8,63 | 120,41±7,89 | 26,59±7,57 |
|  | AL | 43 | M | 117,89±7,98 | 110,55±7,29 | 96,79±10 | 118,06±6,75 | 120,62±7,84 | 28,71±13,14 |
|  |  |  | **P** | ***0,16*** | ***0,32*** | ***0,79*** | ***0,56*** | ***0,67*** |  |
|  | SL | 59 | M | 125,56±7,76 | 113,39±7,08 | 97,97±9,91 | 115,78±9,3 | 124,17±7,28 | 28,83±8,63 |
|  |  |  | **P** | ***0,12*** | ***0,57*** | ***0,61*** | ***0,5*** | ***0,2*** |  |
| Metatarso  Phalangeal | UL | 76 | M | 152,13±9,64 | 159,87±8,96 | 148,15±14,8 | 136,78±10,78 | 149.27±9.79 | 44,6±15,16 |
|  | AL | 43 | M | 154,96±8,05 | 165,7±9,9 | 156,85±17,39 | 141,9±13,11 | 153,98±11,13 | 41,19±12,2 |
|  |  |  | **P** | ***0,49*** | ***0,43*** | ***0,43*** | ***0,06*** | ***0.002*** |  |
|  | SL | 59 | M | 149,9±8,7 | 162,57±7,77 | 153,5±14,47 | 139,29±13,67 | 147,65±10,97 | 41,37±9,89 |
|  |  |  | **P** | ***0,88*** | ***0,79*** | ***0,43*** | ***0,19*** | ***0,92*** |  |

SI 5: Time of occurrence of characteristic events of the stride (expressed as a fraction of cycle duration) and average angles at characteristic events (mean +/-SD). SL: Symmetric loaded, AL: Asymmetric loaded, UL: Unloaded; N: number of unloaded and loaded sequences; * P values <0.05 from the ANOVA analyses; bold shading indicates a significant difference ≤0,05 of ANOVA test. Events of the stride include: initial foot contact (IFC), opposite foot take-off (OTO), opposite foot contact (OFC), take-off of the foot (TO), and final foot contact (FFC).

| **%BW** | **N** |  | **Contact:**  **Y** | **Mid:**  **Y** | **Take off:**  **Y** | **Δh** |
| --- | --- | --- | --- | --- | --- | --- |
| 0 | 51 | M±SD | 1,99±0,2 | 2,09±0,16 | 1,99±0,16 | 0,09±0,05 |
| 15 | 11 | M±SD | 1,94±0,15 | 2,05±0,12 | 1,94±0,12 | 0,1±0,03 |
|  |  | **p** | ***0,4*** | ***0,42*** | ***0,41*** | ***0,4*** |
| 20 | 39 | M±SD | 2,22±0,24 | 2,29±0,19 | 2,17±0,21 | 0,06±0,06 |
|  |  | **p** | ***0*** | ***0*** | ***0*** | ***0,02*** |
| 30 | 11 | M±SD | 2,05±0,07 | 2,12±0,1 | 2,02±0,1 | 0,06±0,06 |
|  |  | **p** | ***0,66*** | ***0,98*** | ***0,77*** | ***0,18*** |
| Total | 61 | M±SD | 2,14±0,24 | 2,21±0,2 | 2,10±0,2 | 0,07±0,05 |
|  |  | **p** | ***0*** | ***0*** | ***0*** | ***0,06*** |

SI 6: Average values, Standard deviations (M±SD) and ANOVA tests values (p values <0.05) for the standardized height of total center of mass (Y), the standardized elevation of total center of mass (Δh) during stance phase according to different %BW in unloaded (%BW=0) and symmetrical loaded. N: number of unloaded and loaded sequences.

| **%BW** | **N** |  | **P1** | **M** | **P2** | **Im** |
| --- | --- | --- | --- | --- | --- | --- |
| 0 | 127 | M±SD | 9,63±0,7 | 7,58±0,59 | 10,2±0,6 | 5,03±0,59 |
| 15 | 8 | M±SD | 10,31±0,46 | 7,38±0,66 | 10,44±0,27 | 4,78±0,61 |
|  |  | **p** | ***0,02*** | ***0,95*** | ***0,55*** | ***0,31*** |
| 20 | 20 | M±SD | 9,98±0,75 | 7,27±0,37 | 9,88±0,47 | 4,74±0,44 |
|  |  | **p** | ***0,06*** | ***0,02*** | ***0*** | ***0,06*** |
| 25 | 8 | M±SD | 8,72±0,51 | 7,52±0,71 | 9,71±0,98 | 4,61±0,96 |
|  |  | **p** | ***0*** | ***0,79*** | ***0,01*** | ***0,08*** |
| 30 | 1 | M±SD | 10,1 | 7,02 | 9,1 | 4,97 |
| Total AL | 37 | M±SD | 9,78±0,85 | 7,34±0,52 | 9,94±0,64 | 4,76±0,59 |
|  |  | **P** | ***0,8*** | ***0,09*** | ***0,02*** | ***0,02*** |

SI 7: Average values, Standard Deviation (M±SD) and values of ANOVA test (p values <0.05) of force parameters (vertical force variables: the first peak (P1); corresponding to the weight acceptance, the second peak (P2) corresponding to the propulsion phase and the minimum point between the two maxima (M) corresponding to the middle of the stance phase and Impulse (Im)) according to different %BW in unloaded (%BW=0) and asymmetrical loaded (AL). N: number of unloaded and loaded sequences.

| **%BW** | **N** |  | **P1** | **M** | **P2** | **Im** |
| --- | --- | --- | --- | --- | --- | --- |
| 0 | 127 | M±SD | 9,63±0,7 | 7,58±0,59 | 10,2±0,6 | 5,03±0,59 |
| 15 | 19 | M±SD | 9,93±0,7 | 7,61±0,64 | 10,07±0,42 | 5,33±0,82 |
|  |  | p | ***0,01*** | ***0,27*** | ***0,79*** | ***0*** |
| 20 | 15 | M±SD | 9,27±0,59 | 7,77±0,31 | 9,63±0,42 | 5,18±0,68 |
|  |  | p | ***0,24*** | ***0,93*** | ***0*** | ***0*** |
| 25 | 42 | M±SD | 9,44±0,95 | 7,25±0,58 | 10,17±0,56 | 4,87±0,59 |
|  |  | p | ***0,06*** | ***0*** | ***0,39*** | ***0*** |
| 30 | 6 | M±SD | 9,54±1,24 | 6,4±0,34 | 9,12±0,77 | 4,32±0,38 |
|  |  | p | ***0,38*** | ***0*** | ***0*** | ***0,02*** |
| Total SL | 82 | M±SD | 9,53±0,88 | 7,37±0,63 | 9,97±0,6 | 4,99±0,7 |
|  |  | p | ***0,2*** | ***0,01*** | ***0*** | ***0,03*** |

SI 8: Average values, Standard Deviation (M±SD) and values of ANOVA test (p values <0.05) of force parameters (vertical force variables: the first peak (P1); corresponding to the weight acceptance, the second peak (P2) corresponding to the propulsion phase and the minimum point between the two maxima (M) corresponding to the middle of the stance phase and Impulse (Im)) according to different %BW in unloaded (%BW=0) and symmetrical loaded (SL). N: number of unloaded and loaded sequences.
